# Profile of Abaloparatide and Its Potential in the Treatment of Postmenopausal Osteoporosis

**DOI:** 10.7759/cureus.1300

**Published:** 2017-05-31

**Authors:** Sri Harsha Tella, Anuhya Kommalapati, Ricardo Correa

**Affiliations:** 1 Endocrinology, Diabetes and Metabolism, National Institute of Health; 2 Internal Medicine, Medstar Washington Hospital Center; 3 NICHD, National Institute of Health

**Keywords:** abaloparatide, teriparatide, bmd, osteoporosis, anabolic agents

## Abstract

Abaloparatide (previously known as BA058) is a synthetic 34-amino acid peptide and novel selective activator of parathyroid hormone receptor 1 (PTHR1) currently under development as a new anabolic agent in the management of osteoporosis. This paper reviews the profile and potential of abaloparatide in the treatment of postmenopausal osteoporosis.

This paper is based on clinical trials and a PubMed search. Search terms used were “abaloparatide”, “BA058”, and “PTHrP”. This review outlines the effects of this anabolic PTHR1 activator, which increases bone mineral density in patients at high risk for osteoporosis. The potential adverse effects of abaloparatide are also summarized.

Abaloparatide has 41% homology to parathyroid hormone (PTH) (1-34) and 76% homology to parathyroid hormone-related protein (PTHrP) (1-34). The molecule was meticulously selected to retain stability and potent bone anabolic activity, and it has a limited effect on bone resorption (hence, a low calcium-mobilizing potential). Abaloparatide has shown promising results in a reduction of new onset vertebral (approximately 86% reduction) and nonvertebral fractures (approximately 43% reduction). In clinical trials to date, abaloparatide appears to have a good safety and tolerability profile with a significantly lower degree of hypercalcemia compared to that of teriparatide. Based on the clinical trials, the optimum dose of abaloparatide is 80 mcg subcutaneous once daily.

## Introduction and background

Bone remodeling is a continuous process by which old bone is replaced by new bone. The normal bone remodeling process consists of five phases: resting, activation, resorption, formation, and reversal. Osteoporosis is a skeletal disease associated with an imbalance between resorption and formation, leading to net loss of bone mass and microarchitecture resulting in the development of fractures. Fractures of the hip and spine are common consequences of osteoporosis that are associated with increased disability and mortality and can impair quality of life in older postmenopausal women. At menopause, an estrogen deficiency causes an increase in osteoclastic resorption without a corresponding increase in osteoblastic anabolic function, leading to a net loss of bone. This process was originally described as “uncoupling.”

Traditional therapies for osteoporosis (e.g., bisphosphonates, selective estrogen receptor modulators, and denosumab) counteract the osteoclastic resorption to help maintain bone mineral density (BMD). Parathyroid hormone (PTH) 1-34, teriparatide, is the first anabolic agent approved by the US Food and Drug Administration (FDA) for the treatment of osteoporosis and is relatively safe and effective in increasing bone mineral density and reducing fracture risk in severe cases of osteoporosis. Though teriparatide has shown great improvements in spine BMD, this effect is seen more evidently in the second year of treatment. Also, its effects on the hip BMD are modest [1-5]. Moreover, studies have shown that teriparatide therapy can lead to a mild degree of hypercalcemia (serum calcium > 10.6 mg/dL) attributed to a mild increase in bone resorption, which also limits the BMD gains notably at cortical sites [1, 4].

Abaloparatide has 41% homology to parathyroid hormone (PTH) (1-34) and 76% homology to parathyroid hormone-related protein (PTHrP) (1-34). It was meticulously selected to retain stability; yet, it has retained a limited effect on bone resorption and, hence, calcium-mobilization [5]. Daily subcutaneous injection of abaloparatide in postmenopausal women with osteoporosis for 24 weeks has demonstrated an increase in the spine, hip, and femoral neck BMD in a dose-dependent manner [5]. It was also shown that abaloparatide has resulted in a greater increase in hip BMD as compared to teriparatide [5]. Our paper outlines the physiology of PTH and PTHrP and the potential utility of abaloparatide (in both the subcutaneous administration and transdermal patches) in the treatment of postmenopausal osteoporosis.

## Review

### Physiological differences in the action of PTH and PTHrP

The molecular mechanisms by which abaloparatide exerts its effects on bone are not fully understood but may be related to the conformation-selective binding of the PTH1 receptor relative to other ligands including PTH and PTHrP [6-9]. Despite sharing some sequence homology, each hormone (PTH and PTHrP) plays a distinct role in bone physiology. One of the key differences between the two hormones is that PTH is secreted by the parathyroid glands and acts in a classical endocrine manner to promote osteoclastic bone resorption and calcium mobilization, whereas PTHrP functions as a paracrine regulatory hormone on bone forming cells. Despite the difference, both hormones act on the parathyroid hormone receptor 1 (PTHR1, a G-protein coupled receptor) by increasing cyclic adenosine monophosphate (cAMP) concentrations. While both hormones act on the same receptor, recent studies have shown that continuous PTHrP administration stimulates bone formation preferentially, whereas continuous administration of PTH leads to more bone resorption than formation [6, 9]. Both abaloparatide and teriparatide showed an increased messenger ribonucleic acid (RNA) expression level for the receptor activator of nuclear factor kappa-B ligand (RANKL) and macrophage colony-stimulating factor in a human osteoblastic cell line; however, these effects were rapidly reversed after the removal of abaloparatide from the culture media, while the effects were sustained in the teriparatide-treated cells [6, 9]. Thus, PTHrP may cause transient stimulation of osteoblast cAMP production, which may result in lower osteoblast-derived RANKL expression and, therefore, less stimulation of bone resorption. Although the molecular mechanisms underlying the differences between abaloparatide and teriparatide are not well understood, the mechanisms may be related to conformational differences based on the affinities of the drugs to the PTHR1 receptor [6-9].

The difference in activity between the two hormones, PTH and PTHrP, may partly be explained by receptor conformational changes named as R0 and RG respectively. It is thought that the actions of PTHrP (1-36) and of abaloparatide were restricted to the cell surface, while PTH (1-34) was more readily internalized and brought about a somewhat more prolonged increase in cyclic AMP in the target cells, even though they increased cAMP in the same cells with comparable potencies [10]. PTH’s longer action can be explained by conformational changes that stabilize the PTH-PTHR1 complex, leading to longer activation of the G protein and persistent cAMP generation [6-7, 11]. This persistence of cAMP generation leads to a more catabolic response, which, in turn, results in elevated calcium levels [8]. In contrast, PTHrP only leads to the transient production of cAMP and, therefore, results in less catabolic action and calcium mobilization relative to its anabolic action, thereby causing a lower degree of hypercalcemia

Similar findings are also demonstrated in a study that evaluated the receptor binding of PTH and PTHrP to PTHR1 [10]. Abaloparatide seems to have greater G-protein binding to a specific conformation (the RG conformation) of the receptor leading to enhanced anabolic activity.

How is Abaloparatide Different from PTHrP?

**Figure 1:**
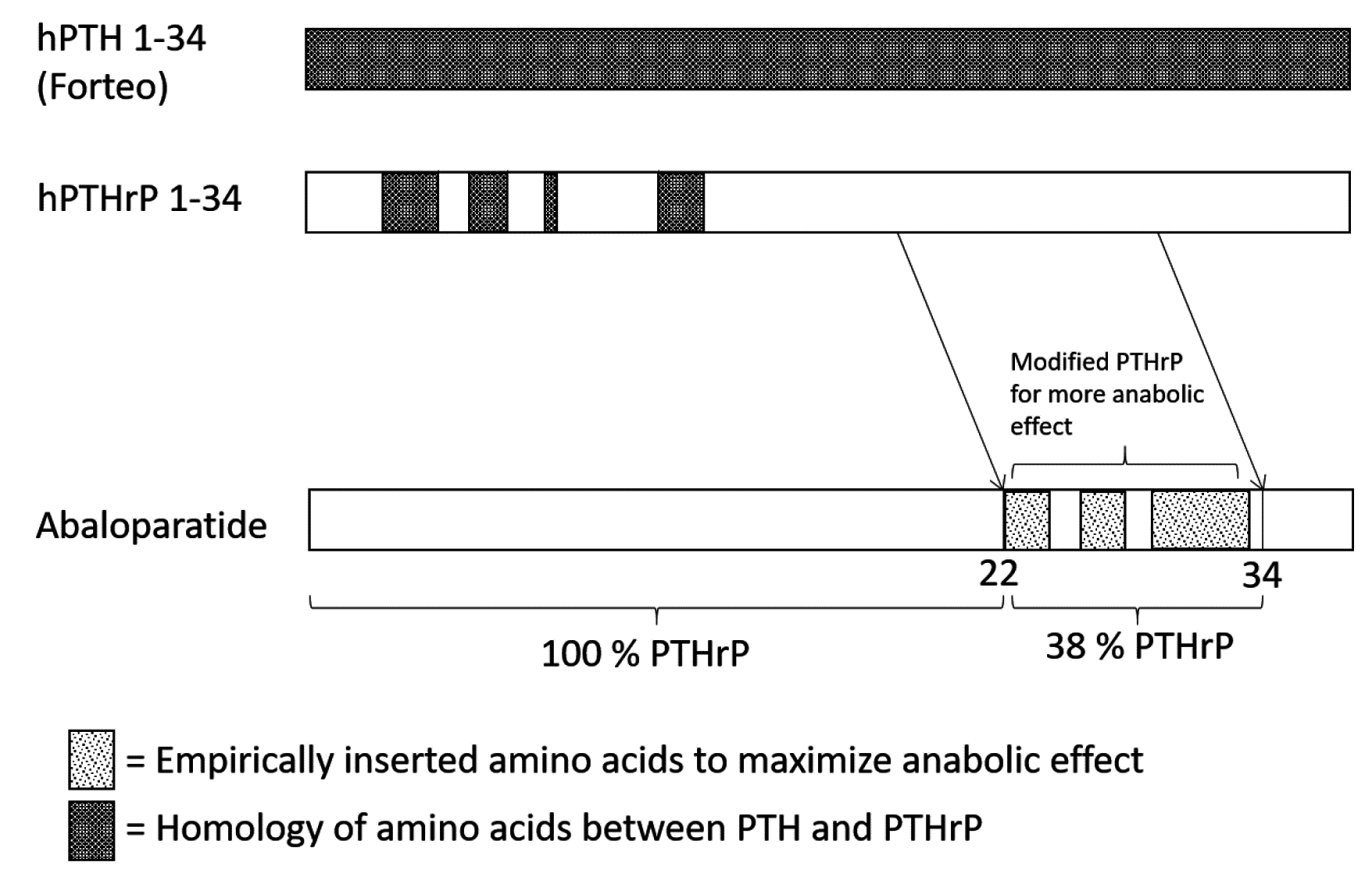
Comparison of Abaloparatide with hPTHrP 1-34 and hPTH 1-34 hPTH 1-34: human parathyroid hormone 1-34; hPTHrP 1-34: human parathyroid hormone-related protein; hPTH 1-34: N-terminal parathyroid hormone 1-34; PTH: parathyroid hormone; PTHrP: parathyroid hormone-related protein

Abaloparatide is an analog of PTHrP that is identical to PTHrP at amino acid residues 1-22, but with multiple substitutions between amino acids 23-34 (38% identical to PTHrP) to maximize the stability of the molecule (Figure 1).

### Pharmacokinetics and pharmacodynamics of abaloparatide 

The drug is being evaluated in clinical trials in two forms: subcutaneous and transdermal. No peer reviewed data have been published as yet about the pharmacokinetic and pharmacodynamic properties of abaloparatide.

Special Populations: Renal Impairment

Abaloparatide was evaluated with post hoc analysis of the Phase 3 Abaloparatide Comparator Trial in Vertebral Endpoints (ACTIVE) clinical trial in patients with mild to moderate renal impairment [12]. No increase in adverse events or decrease in efficacy was noted in this subgroup of subjects despite increased exposure to the drug. No significant increase in BMD was noted in this subgroup compared to patients who had normal renal function. Though there is an increased incidence of hypercalcemia, it is significantly lower than that observed with teriparatide (p = 0.006). Renal computed tomography scans did not show nephrocalcinosis or renal calculi in either of the groups. These results show positive data supporting the use of subcutaneous abaloparatide in patients with mild and moderate renal impairment.

### Effects of abaloparatide on bone tissue

Effects on BMD

In a 24-week randomized trial of 222 treatment-naïve postmenopausal women with osteoporosis, abaloparatide at both 40 and 80 mcg doses significantly increased BMD at the lumbar spine, femoral neck, and total hip compared to that of placebo (p < .001) [5]. The increase in lumbar spine BMD was dose-dependent (in a linear trend) demonstrating 2.9 ± 2.6%, 5.2 ± 4.5%, and 6.7 ± 4.2% increase with 20, 40, and 80 mcg abaloparatide groups, respectively (p < .001). No statistically significant difference in lumbar spine BMD was noticed between the teriparatide group and abaloparatide groups (20, 40, and 80 mcg groups). In contrast, significant improvements in total hip BMD were seen with abaloparatide 40 mcg and 80 mcg as compared to teriparatide (p < 0.047 and p < 0.006, respectively). Abaloparatide, 80 mcg, had superior efficacy in improving total hip BMD compared to that of placebo (p = 0.007). On the other hand, teriparatide failed to demonstrate any significant benefit in improving total hip BMD compared to that of placebo. In this study, abaloparatide (at doses of 40 and 80 mcg) demonstrated superior bone anabolic activity at a cortical site (total hip) compared to teriparatide [5].

One plausible explanation is that abaloparatide has a limited effect on bone resorption resulting in a greater gain in cortical bone mass. This is supported by the fact that there was very little increase in bone resorption markers (collagen type 1 cross-linked C-telopeptide (CTx)) in the first 12 weeks after the initiation of abaloparatide. There was a greater increase in CTx in the teriparatide group compared to that of abaloparatide groups (p < 0.003) [5]. The difference between the abaloparatide and teriparatide may be explained by differing affinities of the two drugs to specific conformations (RO, RG) of PTHR1, as described earlier. Of course, one of the main limitations of the study is having a small number of subjects in each arm (i.e., limiting the power to identify differences between the groups) and changes in volumetric BMD or fracture rate were not measured.

A small subset of subjects (n = 55) had continued the drug for 48 weeks. Although linear increments in BMD were demonstrated with increased doses of abaloparatide, this was not clear for the hip region where BMD increases at 48 weeks were only 2.1% and 2.7% for the 40 mcg and 80 mcg doses, respectively (this is not different from the means at 24 weeks). In contrast, although the magnitude of increases was smaller, total hip BMD did appear to increase further with teriparatide from 24 to 48 weeks. Nonetheless, at 24 weeks, abaloparatide showed a significant increase in hip BMD compared to that of teriparatide. Also, in previous studies, teriparatide was shown to cause intracortical bone remodeling, thus, leading to increased cortical porosity, which is not typically observed with abaloparatide [13-15]. Studies that assess the microarchitecture of bone by invasive (e.g., bone biopsy) or noninvasive studies (e.g., imaging) are required to define the effects of abaloparatide on bone quality, and thus, bone strength (bone strength represents the combined effect of bone density and bone quality).

In a Phase 3 randomized control trial involving 2,463 postmenopausal women (mean age: 69 years; range: 49 to 86 years), abaloparatide was compared with placebo after 18 months of treatment [16]. According to Miller, et al. [17], abaloparatide cohort has demonstrated significant changes (p < .001) in total hip BMD (4.18% vs −0.10%; treatment difference, 4.25% [95% CI, 3.90% to 4.59%]), femoral neck BMD (3.60% vs −0.43%; treatment difference, 4.01% [95% CI, 3.58% to 4.45%]), and lumbar spine BMD (11.20% vs 0.63%; treatment difference, 10.37% [95% CI, 9.75% to 10.98%]) compared to that of placebo. In comparison to teriparatide, the abaloparatide cohort had a greater increase in spine BMD (treatment difference, 1.32 [95% CI, 0.86 to 1.79]; p < .001), femoral neck BMD (1.72% vs 0.87%; treatment difference, 0.81% [95% CI, 0.49% to 1.12%]; p < .001), and total hip BMD (2.32% vs 1.44%; treatment difference, 0.83% [95% CI, 0.58% to 1.08%]; p < .001) at the end of six months. Abaloparatide also showed greater increases in BMD as compared to teriparatide at the total hip and femoral neck at 12 and 18 months (p < .001), while it showed greater efficacy at the spine at 12 months (p < .001) but not at 18 months (p = 0.17). The magnitude of these increases in BMDs at lumbar spine and hip seems to be robust when compared with currently available therapies.

Abaloparatide is also currently being evaluated in a transdermal delivery system. A pilot study showed promising results in approximately 200 postmenopausal women who received a short wear-time abaloparatide transdermal patch coated with doses of 50 µg, 100 µg, and 150 µg, using solid Microstructured Transdermal System (3M, USA) which consists of an array of 316 microprojections that penetrate the skin to about 250 µm into the upper dermis [18]. At the end of 24 weeks, there was a dose-dependent increase in both spine BMD and total hip BMD as compared to control patches (p < .001 at both sites). However, the increase in BMD is slightly less than that observed with the subcutaneous formulation. It remains to be determined if the transdermal formulation results in a reduction of vertebral fractures while maintaining better safety and tolerability profiles.

Effect on Bone Turnover Markers (BTMs)

In a 24-week randomized trial of 222 treatment-naïve postmenopausal women with osteoporosis, abaloparatide, 40 mcg and 80 mcg, significantly increased bone formation markers (serum type 1 procollagen (P1NP) and osteocalcin) as early as one week, and the effects showed a significant dose response (linear trend; p < .001) [5]. At 24 weeks, all doses of abaloparatide (20 mcg, 40 mcg, and 80 mcg doses) showed a statistically significant increase in bone formation markers as compared to placebo (p < .001). However, teriparatide demonstrated superiority in increasing the bone formation markers when compared to the 20 mcg abaloparatide group (p < .001), although the increase was not significantly different when compared to abaloparatide at 40 mcg and 80 mcg doses. Bone resorption markers did not show any uptrend in the abaloparatide group until 12 weeks of therapy. Moreover, CTx (a bone resorption marker) was significantly higher in the teriparatide group than in any of the abaloparatide groups (p < .003). No linear trend in CTx elevation was noted at higher doses of abaloparatide. In short, both formation and resorption markers were higher in response to teriparatide. The difference in BMD (favoring the abaloparatide group) may be explained by a lower resorption in response to abaloparatide in postmenopausal women.

Similar positive results were seen in an 18-month randomized, multicenter placebo and teriparatide controlled trial in 2,463 postmenopausal women with osteoporosis [17]. In this study, both abaloparatide, 80 mcg, and teriparatide, 20 mcg, showed a significant increase in both bone formation and resorption markers (p < .001). However, abaloparatide and teriparatide differed in the pattern of bone turnover marker changes. The increase in CTx was much less in the abaloparatide group (46%, 56%, and 69% less at six, 12, and 18 months, respectively; p < .001 for all months), while the differences in the formation marker between abaloparatide and teriparatide were much closer (-12% at six months, -28% at 12 months, and -33% at 18 months; p < .001 for all months), favoring net bone formation with abaloparatide when compared with teriparatide.

Effects on Fractures

The efficacy and safety of abaloparatide in treating vertebral fractures was evaluated in a multicenter randomized control Phase 3 trial (ACTIVE) involving 2,463 postmenopausal women (mean age: 69 years; range: 49 to 86 years) [17]. The main inclusion criteria for the study were postmenopausal women with BMD T-scores < −2.5 and > −5.0 at the lumbar spine or femoral neck and radiological evidence of mild or moderate lumbar or thoracic vertebral fractures or a history of low-trauma nonvertebral fracture within the past five years. The subjects were divided into three arms composed of blinded daily subcutaneous injections of placebo (n = 821); blinded 80-μg abaloparatide (n = 824); or open-label 20 μg teriparatide (n = 818) for 18 months.

According to Miller, et al, in the abaloparatide group, new morphometric vertebral fractures occurred in 0.58% (n = 4) of participants compared to that of placebo (4.22%; n = 30) with a risk difference (RD) of −3.64 (95% CI, −5.42 to −2.10) and a relative risk of 0.14 (95% CI, 0.05 to 0.39) [17]. The authors concluded abaloparatide resulted in a statistically significant reduction of vertebral fractures compared to placebo (p< .001). The Kaplan-Meier estimated event rate for nonvertebral fracture was significantly lower (p = 0.049) in the abaloparatide group (2.7%) as compared to that of placebo (4.7%; RD of −2.01 (95% CI, −4.02 to −0.00); hazard ratio (HR), 0.57 (95% CI, 0.32 to 1.00)). Similar promising results were seen in the teriparatide group when compared to that of placebo. Moreover, no statistically significant difference was seen between the teriparatide and abaloparatide groups at the end of 18 months in terms of vertebral fractures.

However, the results for major osteoporotic fractures were in favor of abaloparatide when compared to that of placebo or teriparatide. In terms of time to event for major osteoporotic fractures for abaloparatide vs placebo, the HR was 0.30 (95% CI, 0.15 to 0.61; p < .001), and for teriparatide vs placebo, the HR was 0.67 (95% CI, 0.39 to 1.14; p = 0.14), demonstrating superiority of abaloparatide in decreasing time to event of major osteoporotic fractures (log rank p-value < .001 for abaloparatide vs placebo, 0.03 for abaloparatide vs teriparatide).

### Safety and tolerability

Abaloparatide has shown a favorable safety profile in Phase 2 and Phase 3 trials and was well tolerated. In a 24-week randomized Phase 2 trial of 222 treatment-naïve postmenopausal women with osteoporosis, abaloparatide was well tolerated with no clinically meaningful differences in treatment-associated adverse events when compared to placebo and teriparatide groups [5]. The incidences of headache and dizziness were slightly elevated in the abaloparatide group. Most adverse events were classified as mild to moderate by the authors. Abaloparatide had a significantly lower incidence of hypercalcemia (i.e., calcium levels > 10.5 mg/dL) as compared with teriparatide (p < 0.01). No immune-mediated adverse events were reported with abaloparatide.

In a Phase 3 trial comparing abaloparatide with placebo and an open-label arm of teriparatide, the study drug was comparable to placebo and teriparatide in terms of serious treatment-emergent adverse events [17]. The most common adverse events that led to study drug discontinuation were nausea (1.6%), dizziness (1.2%), headache (1.0%), and palpitations (0.9%), which were all generally mild to moderate in severity. Abaloparatide demonstrated superiority over teriparatide in terms of incidence of hypercalcemia. In the Phase 3 study [17], abaloparatide led to hypercalcemia at a significantly lower rate than teriparatide (3.4 vs. 6.4%; RD, −2.96 (95% CI, −5.12 to −0.87); p = 0.006) at any time point. Also, abaloparatide produced no increase in cardiovascular events associated with hypercalcemia.

Table 1 summarizes the pivotal trials of subcutaneous abaloparatide

**Table 1:** Summary of Pivotal Trials of Subcutaneous Abaloparatide Abbreviations: BMD: bone mineral density; CI: confidence interval; CTx: collagen type 1 cross-linked C-telopeptide; P1NP: serum type 1 procollagen; RD: risk difference.

Author/Trial	Study drugs	Endpoints (Primary and Secondary)	Safety and Tolerability
Leder, et al. [5], N=222 postmenopausal women	Abaloparatide, 20, 40, or 80 μg, or teriparatide, 20 μg, for 24 weeks.	There was a 2.9%, 5.2%, 6.7%, and 5.5% increase in lumbar spine BMD in the abaloparatide (20-, 40-, 80-μg) and teriparatide groups, respectively. The total hip increases in the 40- and 80-μg abaloparatide groups were greater than both placebo (0.4%) and teriparatide (0.5%).	Treatment-associated adverse events are similar among all the groups. Abaloparatide, 80 mcg group, experienced slightly elevated dizziness events compared to that of other treatment arms. Abaloparatide cohorts (all doses) had lower hypercalcemia at 4 hours compared to that of teriparatide group H (p < .01).
Cosman, et al. [16], N = 2,463 postmenopausal women	Abaloparatide, 80 mcg, teriparatide, 20 mcg, and placebo.	Increase in CTx was much less in abaloparatide group (-46% at 6 months, -56% at 12 months and -69% at 18 months; p < 0.001 at 6, 12, and 18 months). In terms of P1NP, abaloparatide was much closer to teriparatide (-12% at 6 months, -28% at 12 months, and -33% at 18 months; p < 0.001 at 6, 12, and 18 months). Compared to placebo, abaloparatide arm demonstrated significant changes (p < 0.001) from baseline BMD at the total hip, femoral neck, and lumbar spine (all p < 0.001). The increase in BMD was greater in the abaloparatide arm at the total hip and femoral neck at 6, 12, and 18 months (p < .001) and at the lumbar spine at 6 and 12 months (p < .001). Compared to placebo, abaloparatide arm demonstrated significant changes (p < 0.001) from baseline BMD at the total hip, femoral neck, and lumbar spine (all p < 0.001).	NA
Miller, et al. [17], N = 2,463 postmenopausal women	Abaloparatide, 80 mcg, teriparatide, 20 mcg, and placebo.	Abaloparatide demonstrated superiority in BMD increase over placebo at all sites (all p < .001). The Kaplan-Meier estimated event rate for nonvertebral fracture was significantly lower (p = 0.049) in abaloparatide group (2.7%) compared with placebo (4.7%) Abaloparatide demonstrated superior efficacy in reducing nonvertebral fractures.	Abaloparatide cohort had lower incidence of hypercalcemia (3.4%) vs teriparatide (6.4%; RD, −2.96 [95% CI, −5.12 to −0.87]; p = 0.006). Mild to moderate nausea (1.6%), dizziness (1.2%), headache (1.0%), and palpitations (0.9%) lead to abaloparatide discontinuation.

### Update

On April 28, 2017, abaloparatide was approved by the FDA for subcutaneous injection for postmenopausal women with osteoporosis at high risk for fracture due to a history of osteoporotic fracture, multiple risk factors for fracture, or patients who have not responded to or are intolerant to other available osteoporosis therapy. The recommended dose is 80 μg administered subcutaneously once daily into the periumbilical region of the abdomen. Abaloparatide use is not recommended in patients who are at increased risk of osteosarcoma, Paget's disease of the bone or unexplained elevations of alkaline phosphatase, open epiphyses, bone metastases or skeletal malignancies, hereditary disorders predisposing to osteosarcoma, or prior external beam or implant radiation therapy involving the skeleton. Also, abaloparatide use is not recommended in individuals who have already used other parathyroid hormone analogs (e.g., teriparatide) for two years.

## Conclusions

Abaloparatide has the potential to reduce both nonvertebral and vertebral fractures and to improve BMD in postmenopausal women with osteoporosis, regardless of age, prior fracture history, or BMD score, with a well-tolerated side effect profile. Based on the pivotal trials, abaloparatide has demonstrated noninferiority to teriparatide regarding the increase in BMD at the lumbar spine and the reduction of vertebral fracture risk, whereas it has demonstrated a greater increase in BMD at the femoral sites. Nonetheless, it is important to confirm this by iliac crest histomorphometry and other noninvasive tests of bone strength like finite element analysis, particularly for the cortical bone. Moreover, further studies are needed to determine the long-term safety and efficacy of abaloparatide administration after a course of antiresorptive therapy (e.g., denosumab or bisphosphonates). An anabolic agent with less resorptive stimulation might address the unmet needs of a large group of osteoporotic patients who warrant more potent anti-osteoporotic treatment.
